# Iatrogenic Spinal Deformity Following Spinal Intradural Arachnoid Cyst Fenestration Despite Minimal Access With Laminoplasty and Endoscopy in a Pediatric Patient

**DOI:** 10.7759/cureus.22053

**Published:** 2022-02-09

**Authors:** Josue D Ordaz, Andrew Huh, Virendra Desai, Jeffrey S Raskin

**Affiliations:** 1 Neurological Surgery, Indiana University School of Medicine, Indianapolis, USA; 2 Neurological Surgery, University of Oklahoma Health Sciences Center, Oklahoma City, USA

**Keywords:** iatrogenic scoliosis, spinal cyst, arachnoid cyst, ultrasound, endoscopy, multimodal imaging

## Abstract

Spinal intradural arachnoid cysts (SAC) are non-neoplastic lesions that can cause spinal cord compression and present with myelopathy, radiculopathy, and/or back pain. Because these cysts typically span multiple levels, endoscopy could be a useful tool to avoid wide exposure. We present an 8-year-old patient with a history of gait imbalance and urinary incontinence who was found to have a SAC spanning C7 to T6 causing spinal cord compression. An osteoplastic laminoplasty was performed from T4 to T7 followed by ultrasonic verification of intracystic septations, dural opening, and cyst fenestration. A flexible endoscope was then introduced into the cystic cavity to guide complete rostral and caudal decompression of the arachnoid cyst. At six months follow-up, the patient was able to ambulate independently, but his urinary incontinence remained unchanged. Despite the combination of ultrasound and neuroendoscopy to minimize exposure, our patient suffered from worsening kyphosis from 36 degrees preoperative to 55 degrees postoperative and worsening scoliosis from 17 to 39 degrees which required treatment with a thoracolumbar sacral orthosis. Preoperative imaging demonstrated a reverse S-shaped scoliosis with the apex at T6 and T7 which were the levels included in the laminoplasty. This illustrates the need for careful preoperative risk stratification to avoid this postoperative complication.

## Introduction

Spinal intradural arachnoid cysts (SACs) are expansile lesions of the arachnoid space which can cause spinal cord compression [1]. They are mostly considered congenital or idiopathic lesions but may be acquired through posttraumatic [2], surgical [3], or inflammatory etiologies [4]. Modern imaging frequency commonly identifies incidental and asymptomatic SACs; however, the most common presenting signs and symptoms are hypertonia, weakness, back pain, and paresthesia [1,5-7]. Surgical management is preferred for decompression of the spinal cord via fenestration, extirpation, or shunting, with access via laminoplasty or laminectomy.

SACs are most frequently present in the thoracic spine and are less commonly identified in the cervical and lumbar spine [5]. They usually span multiple vertebral levels requiring multilevel contiguous or skip laminoplasties which increases morbidity and the risk of postsurgical kyphosis [8]. Minimal access surgery is attractive to decreased morbidity and improves surgical recovery; however, limited access risks partial decompression and surgical failure due to septations outside of the surgical view or lack of a sufficient channel for fluid egress. Endoscopy, a minimally invasive modality to treat a myriad of conditions, may mitigate the risk of surgical failure by maximizing visualization within the spinal canal. Recently, a retrospective literature review on endoscopic treatment of spinal arachnoid cyst in adults demonstrated improvement in symptoms in six out of nine patients without complications [9].

We present a pediatric patient with a multilevel cervicothoracic arachnoid cyst spanning C7 to T7, causing ataxia and urinary incontinence. We performed a fluoroscopic guided T4 to T7 osteoplastic laminoplasty, followed by ultrasound verification, and then flexible endoscopic-assisted cyst fenestration and confirmation.

## Case presentation

**Figure 1:**
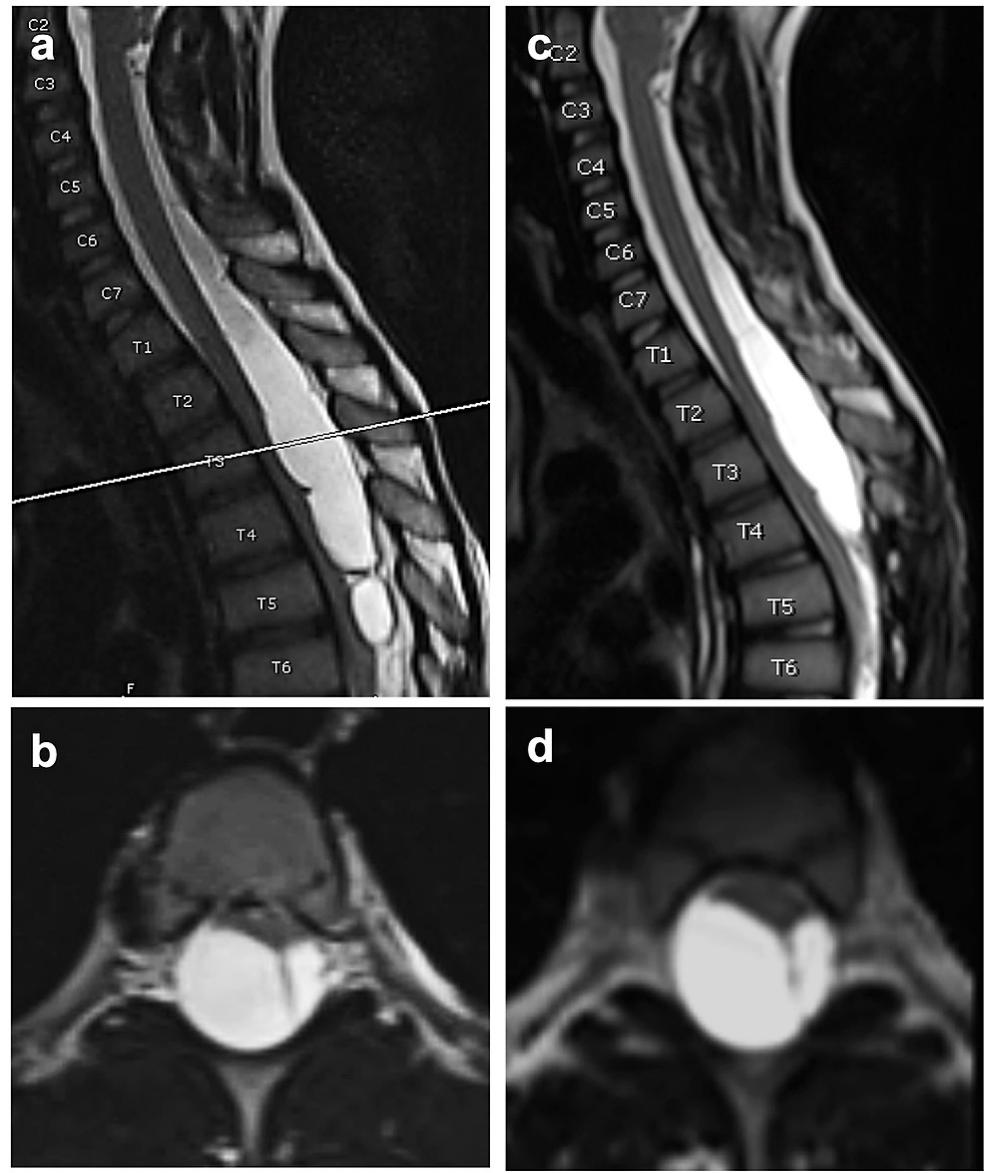
Preoperative and postoperative MRI. (a and b) Preoperative MRI demonstrating arachnoid cyst with significant mass effect on the spinal cord. (c and d) Three-month postoperative MRI demonstrating unchanged cyst and mass effect on the spinal cord.

An 8-year-old male with a history of lumbosacral myelomeningocele and shunted hydrocephalus presented with two months of ataxic gait with inability to ambulate independently and worsening urinary incontinence with frequent and incomplete bladder voids despite botox and muscarinic agonist. An MRI of the spine demonstrated a non-enhancing cystic lesion extending from C7 to T7 with severe cord compression suggestive of an intradural arachnoid cyst which measured 9.2 cm in craniocaudal and 1.5 cm x 1.3 cm in the transverse dimensions (Figure 1). Thus, he was indicated for a T4 to T7 osteoplastic laminoplasty and cyst fenestration.

**Figure 2:**
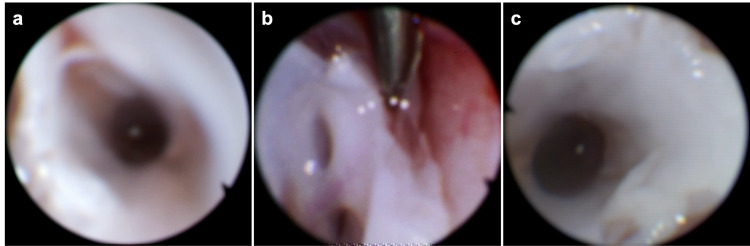
Endoscopic view within the cyst. (a-c) Dark cavity rostrally and caudally within the cyst demonstrating free flow of CSF.

A fluoroscopic guided incision and bony localization were performed followed by T4 to T6 laminoplasty. A standard ultrasound probe depicted bony removal above and below the entire extent of the thick-walled capsule and membranes identified on preoperative imaging. Following dura opening, the arachnoid cyst was incised and cerebrospinal fluid (CSF) egressed under high pressure. Intracystic flexible endoscopy demonstrated additional caudal and rostral septations which prompted wider bony removal and cyst fenestration until a dark cavity and free-flowing CSF were visualized with the endoscope (Figure 2).

**Figure 3:**
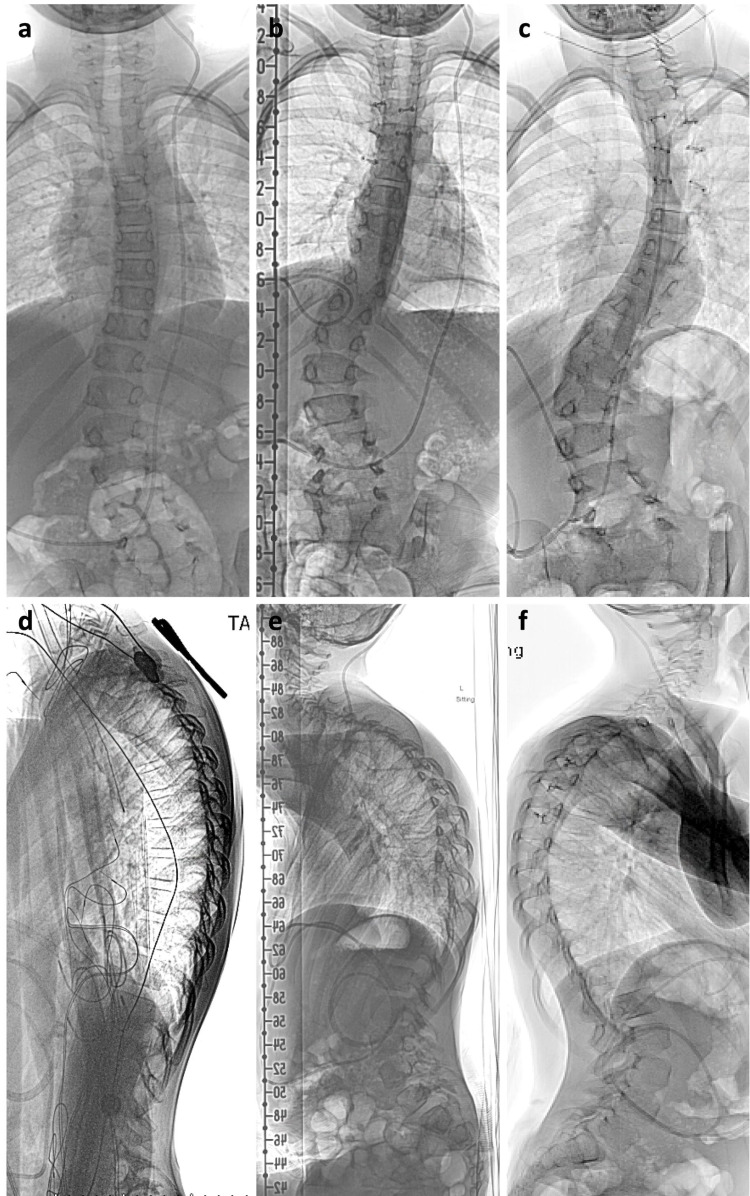
Postoperative kyphoscoliosis. (a, d) Preoperative anteroposterior (AP) and lateral films demonstrated reverse S-shaped scoliosis. (b, e) Three-month postoperative scoliosis films with worsening kyphoscoliosis. (c, f) Six-month postoperative scoliosis films demonstrating progressive scoliosis but stable kyphosis.

At one month follow-up, the patient’s gait had significantly improved. He was walking and running independently, but his urinary incontinence was unchanged from preoperative. An MRI at three months demonstrated worsening thoracic kyphosis without change in cystic mass (Figure 1). Thus, standing scoliosis films were obtained which showed reverse S-shaped scoliosis and kyphosis measuring 39 and 55 degrees, respectively (Figure 3). A thoracic lumbar sacral orthosis (TLSO) brace was prescribed to halt the progression of kyphoscoliosis until the patient reached skeletal maturity. Standing scoliosis films were repeated at six months postoperative which demonstrated worsening reverse S-shaped scoliosis but stable kyphosis. Despite this, the patient continued to show benefit from surgery since he was still able to mobilize without assistance.

## Discussion

Conventional treatment of SACs includes excision, fenestration, or cysto-peritoneal shunting via open laminectomy or laminoplasty. No surgical guidelines exist and specific treatment is tailored based on the location, size, and morphology of the SAC. All three techniques have shown efficacy in the literature [1,6,7,10] (Table 1). A recent retrospective review found similar results for both excision and fenestration groups [7]. Four patients underwent cysto-peritoneal shunt, 48 had cyst fenestration, and 18 had resections. Seven patients required additional cyst wall resection after 1.5-31 months due to insufficient cyst shrinking and persistent symptoms. The mode of treatment was not predictive. Most of the reoperated cysts were multiseptated and posthemorrhagic, which they found was a risk factor. Thus, it could be beneficial to use endoscopy to verify complete fenestration of SAC to prevent reoperation.

**Table 1:** Location of SACs and presenting signs and symptoms published in the literature.

		Percentage (%)			
		Osenbach et al. 1992 [1]	Moses et al. 2018 [5]	Bond et al. 2012 [6]	Schmutzer et al. 2020 [7]
Location	Cervical	21	19	27	14
	Thoracic	64	90	72	63
	Lumbar	14	10	33	24
Common symptoms	Back pain	93	57	32	92
	Parasthesias	64	67		64
	Weakness	64	67	39	80
	Radiculopathy	35		13	-
	Urinary dysfunction	43	24	7	36
	Gait impairment	21	52	32	80
Common signs	Sensory loss	85	-	10	-
	Hypertonia	72	-	19	-
	Hyperreflexia	64	-	-	-
	Motor deficit	64	-	-	-

In our opinion, the benefit of using endoscopy for treatment of SACs is two-fold: first, to verify complete fenestration especially in multilevel arachnoid cysts, which could avoid reoperation; and second, to minimize the surgical exposure, which could theoretically reduce postoperative kyphosis. It may seem high-risk to place an endoscope in the spinal subarachnoid space. However, SACs provide a larger space than normal which is amenable to the flexible endoscope’s diameter. In this case, the cyst transverse dimension was 1.5 x 1.3 cm. A flexible endoscope was used which had a distal tip outer diameter of 2.8 mm and working channel within it. Thus, the arachnoid cyst provided adequate space for safe use of the endoscope. However, it is critical to have continuous irrigation through the endoscope to avoid collapse of the cyst during CSF egress.

We performed a laminoplasty to reduce the risk of postoperative kyphosis. However, postoperative imaging demonstrated worsening kyphoscoliosis. Some studies have found no difference in progressive spinal deformity in patient with laminoplasty versus laminectomy [11]. Looking back at our patient’s preoperative AP films, we observed a levoscoliosis curve with apex at T6 and T7, which were included in our laminoplasty procedure (Figure 2). Studies have found that laminectomy at the apex of a scoliosis deformity can result in iatrogenic progressive deformity [12]. Moreover, natural history studies on progressive degenerative scoliosis have shown more rapid progression of scoliosis after laminectomy at index level [13]. The mechanism involves disruption of the posterior tension band (PTB) and thoracic postural paraspinous musculature [12,14,15]. Further increasing his risk were preoperative deformity and age <18; both shown to be independent risk factors for developing postoperative deformity [14,15]. We believe this deformity could have been avoided by posterior instrumented fusion or through a minimally invasive approach with tubular retraction system to minimize disruption of the PTB, though this could be technically challenging to treat a SAC spanning C7 to T7.

## Conclusions

Our patient developed iatrogenic kyphoscoliosis following spinal intradural arachnoid cyst (SAC) fenestration despite minimal access surgery. Endoscopy and ultrasound are useful technologies to treat SACs that span multiple levels. Though these tools may help reduce surgical exposure, preoperative evaluation for kyphoscoliosis with clinical examination and/or scoliosis films should be done before performing multilevel laminectomy or laminoplasty in the pediatric population to reduce the risk of postoperative worsening spinal deformity.
